# Femtosecond Laser Microprinting of a Polymer Optical Fiber Interferometer for High-Sensitivity Temperature Measurement

**DOI:** 10.3390/polym10111192

**Published:** 2018-10-26

**Authors:** Chi Li, Changrui Liao, Jia Wang, Zongsong Gan, Yiping Wang

**Affiliations:** 1 Key Laboratory of Optoelectronic Devices and Systems of Ministry of Education and Guangdong Province, College of Optoelectronic Engineering, Shenzhen University, Shenzhen 518060, China; lichi2017@email.szu.edu.cn (C.L.); wangjia2016@email.szu.edu.cn (J.W.); ypwang@szu.edu.cn (Y.W.); 2 Wuhan National Laboratory for Optoelectronics (WNLO), Huazhong University of Science and Technology (HUST), Wuhan 430074, China; ganzongsong@hust.edu.cn; 3 Huazhong University of Technology Shenzhen Research Institute, No. 9 Yue Hing Third Road, Nanshan District, Shenzhen 518057, China

**Keywords:** femtosecond laser microprinting, fiber interferometer, temperature measurement

## Abstract

Femtosecond laser induced multi-photon polymerization technique can be applied to fabricate an ultracompact polymer optical fiber interferometer which was embedded in a section of hollow core fiber. The production of the photoresin, used in this work, is described. Such a device has been used for temperature measurement, due to its excellent thermal properties. Transmission spectrum, structural morphology, and temperature response of the polymer optical fiber interferometer are experimentally investigated. A high wavelength sensitivity of 6.5 nm/°C is achieved over a temperature range from 25 °C to 30 °C. The proposed polymer optical fiber interferometer exhibits high temperature sensitivity, excellent mechanical strength, and ultra-high integration. More complex fiber-integrated polymer function micro/nano structures produced by this technique may result in more applications in optical fiber communication and optical fiber sensors.

## 1. Introduction

Multiphoton polymerization (MPP), induced by femtosecond (Fs) laser, is a novel 3D microprinting technology. This method therefore pushes the ongoing trend of miniaturization forwards. Direct laser writing, with highly transparent photoresists, enables 3D microprinting to enter the realm of manufacturing optical elements at the micro/nanometre scale [1,2,3,4]. Thus, this precise fabrication of complex optical elements on demand becomes possible, such as microfluidic devices, micro-electromechanical systems (MEMS), and metamaterials [5,6,7]. Fiber interferometer, which has advantageous of excellent strain, temperature sensitivity, and compact structure, is another reliable fiber sensor configuration, compared to fiber Bragg grating (FBG) [8,9]. However, traditional silica fiber interferometers are limited by its low thermal expansion coefficient (~5.5 × 10^−7^/°C) and therefore cannot achieve high temperature sensitivity (typically ~10 pm/°C for silica fibers) [10,11]. Polymer optical fiber interferometers (POFI) with high thermal expansion coefficient (~10^−4^/°C) of the polymer material that composes the waveguide are considered as a better alternative for high sensitivity temperature sensor [12]. In 2009, Dong C.H. et al. provided a high-Q polydimethylsiloxane optical microsphere for thermal sensing, and it achieved a sensitivity of 245 pm/°C [13]. In 2017, Han Y.G. reported on a polymer-overlaid Microfiber Mach-Zehnder interferometer and achieved a temperature sensitivity of 18.3 pm/°C [14].

In this communication, we report an ultracompact POFI microprinted by Fs laser in a silica hollow core fiber for temperature measurements [15]. The output will generate mode interference in one certain polarization input, due to rectangular waveguide [16]. The temperature sensitivity of ~6.4 nm/°C was achieved over the temperature range from 25 °C to 30 °C and the maximum operating temperature of this device is found to be 80 °C. This POFI exhibits a high temperature sensitivity, high mechanical strength and ultracompact fiber-integrated structure. As such, the device may be beneficial for high-precision biological temperature measurements, and this fabrication method presents a new generation of research for complex polymer fiber-integrated device [17,18].

## 2. Materials and Methods

**Figure 1:**
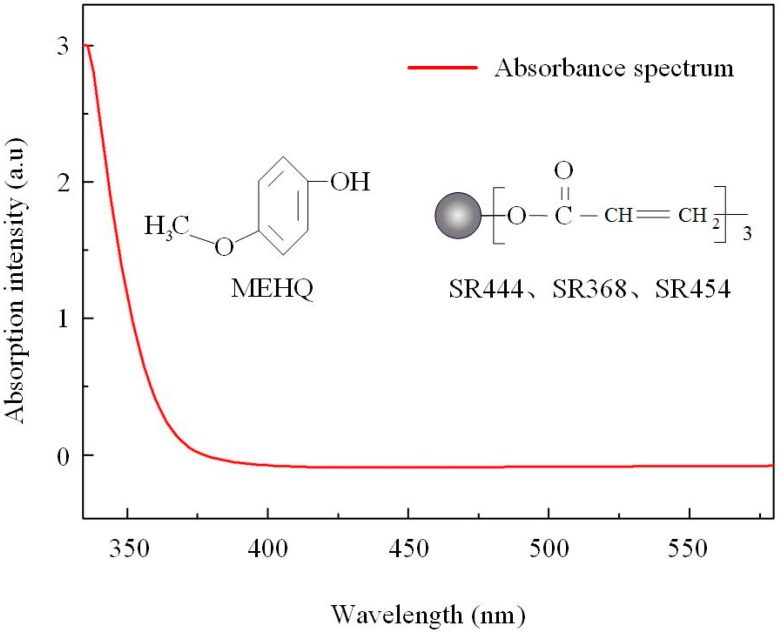
The single-photon absorbance spectrum of the photoresin used in this work. The monomers used in the photoresin are SR368, which was used to improve the chemical reactivity for high photosensitivity and mechanical strength against the damage and the shrinkage during the developing process, and SR444, which was introduced to keep the viscosity from decreasing the photoinhibition efficient, and SR454, which was used to improve the mechanical strength of the photoresin.

Generally speaking, polymethyl methacrylate (PMMA) and polydimethylsiloxane (PDMS) are typical materials for polymerization [19]. However, these solid photoresin are not suitable for fabricating fiber-integrated polymer micro-structure, thus a liquid photoresin with better mechanical property has been investigated. The photoresin used in this work is prepared, as follows: Photo-initiator (IGR-369, Ciba-Geigy) with a mole ratio of 2.5%, trifunctional monomer SR444 (Pentaerythritol Triacrylate, from Sartomer, Figure 1) with a mole ratio of 40%, trifunctional monomer SR368 (Tris (2-Hydroxy Ethyl) Isocyanurate Triacrylate, from Sartomer, Figure 1) with a mole ratio of 30%, trifunctional monomer SR454 (Ethoxylated3 Trimethylolpropa-ne Triacrylate, from Sartomer, Figure 1) with a mole ratio of 25%, were first mixed and dissolved in acetone. To guarantee sufficient dissolve, the mixture was heated at 50 °C for 1 h. Then, 4-Hydroxyanisole (MEHQ, from Sigma Aldrich, Saint Louis, MI, USA, Figure 1) used for polymerization inhibitor was added in the mixture with a mole ratio of 0.5%. After a complete dissolving of MEHQ, the mixture was heated to 120 °C. Then, tetraethylthiuram disulphide (TED, from Sigma Aldrich) with a mole ratio of 2.0% was adopted as an accelerator promoter and the mixture was kept at 120 °C for at least 10 mins. A centrifuge is used to remove any undissolved chemicals. The mixture is vacuumed to remove residual acetone. The final photoresin is heated at 60 °C for 1 h. SR368 was used to improve the chemical reactivity for high photosensitivity and mechanical strength against the damage and the shrinkage during the developing process. SR444 was introduced to keep the viscosity from decreasing the photoinhibition efficient. SR454 was used to improve the mechanical strength of the photoresin. TED was used to further improve its mechanical strength [20]. The single-photon absorbance spectrum of the photoresin was shown in Figure 1.

**Figure 2:**
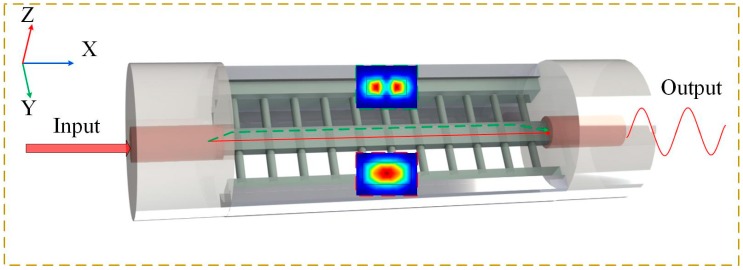
Schematic diagram of the polymer optical fiber interferometer (POFI) microprinted by femtosecond (Fs) laser in a silica hollow core fiber and two fiber modes created interference in the right-TCF.

As illustrated in Figure 2, a polymer optical fiber (POF) is microprinted in a silica hollow core fiber, which has been opened by Fs laser ablation. Both ends of the POF are stably connected with two pieces of thin-core fibers (TCFs). A pair of bases are microprinted to improve to the connection strength of polymer structure with quartz wall, while the jacket tube protects the polymer structure from external contaminations and makes the device much more robust compared with a bare air-cladding microfiber. The POF is suspended by means of a grating structure to provide a contamination-free platform for the light-matter interaction through the evanescent field. Due to the rectangular size of the POF, high-order modes mainly exist in Y direction. In Figure 2, the dotted and solid lines denote the high-order and fundamental modes, respectively. Two kinds of mode recombine at right-TCF, resulting in a interference pattern. The output intensity of POFI can be described as [21]:

(1) $$I=I_{1}+I_{2}+2\sqrt{I_{1}I_{2}}\cos\left(2\pi \Delta \left(\mathrm{nL}\right)/\lambda \right),$$

while the $I_{1}$ and $I_{2}$ represent the beam power of fundamental mode and high-order mode, λ is the wavelength, and Δ(nL) is the optical path difference (OPD) between two interference beams. The interference dip appears at the wavelength:

(2) $$\lambda_{\mathrm{dip}}=2\Delta \left(\mathrm{nL}\right)/\left(2m+1\right),$$

where m is an integer. The free spectral range (FSR) of the interference fringe dip is determined by OPD, as:

(3) $$\mathrm{FSR}=\lambda^{2}/\left(\Delta \left(\mathrm{nL}\right)\right).$$

**Figure 3:**
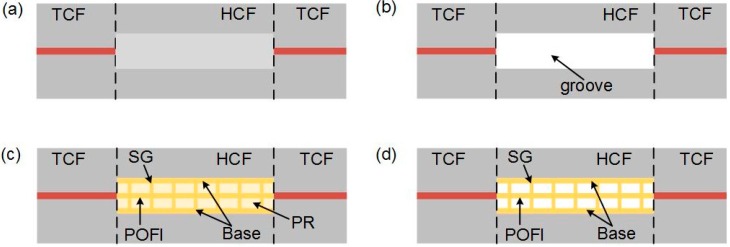
(**a**) A hollow core fiber (HCF) was spliced between two thin-core fibers (TCFs), and (**b**) the HCF was opened by Fs laser ablation. (**c**) The designed POFI structure was microprinted in the HCF using MPP. (**d**) The residual liquid photoresin (PR) in the HCF was cleaned using acetone.

The device fabrication mainly follows four-step processes: As Figure 3a shows, a section of hollow core fiber (internal/external diameter of 30/120 μm, HCF) is spliced between two TCFs, by using optimized splicing parameters (−10 bit for 400 ms in FUJIKURA 80S fiber fusion splicer). Then, as shown in Figure 3b, the middle of HCF was directly opened by focused Fs laser beam with a 20× objective (NA = 0.4). The Fs laser used in this step has 800 nm center wavelength and 1 kHz repetition rate. The laser power before entering the objective was adjusted to be 6 mW. After washing out the residual debris generated in ablation step, the opened HCF was filled with self-made photoresin (PR). Then, the designed POFI structure was microprinted in the HCF by Fs laser induced multi-photon polymerization. As shown in Figure 3c: Firstly, a pair of bases were polymerized along the inner HCF surface to enhance cohesive strength, between the HCF and the polymer micro-structure, with a 50× objective (NA = 0.7) and a speed of 4 mm/s. Then, the suspended POF held by a series of supported grateing (SG) were polymerized by a 63× oil objective (NA = 1.4) and a speed of 0.2 mm/s. The polymer structure was printed in the hollow core fiber by use of a line-by-line and layer-by-layer scanning method. The Fs laser used in this step has 1026 nm center wavelength, 220 kHz repetition rate, and 250 fs pulse width and the power before entering the oil objective was adjusted to 1.80 mW, thus the intensity/irradiance in W/cm^2^ per pulse for polymerization can be calculated as 3.28 × 10^12^ W/cm^2^ [22]. Finally, the sample was immersed in acetone for 30 min to clean the residual liquid PR and Figure 3d shows the final diagram [23].

## 3. Results

**Figure 4:**
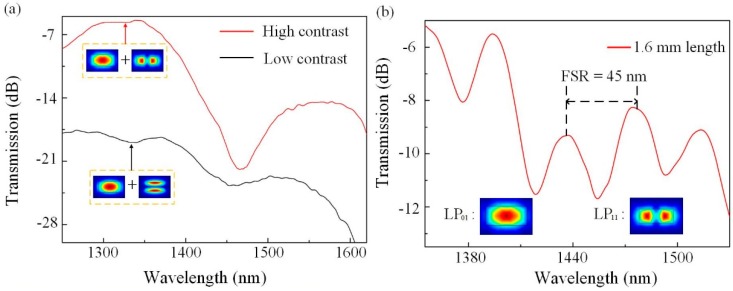
(**a**) The spectral comparison of one POFI with different polarization input. (**b**) Transmission spectrum of one POFI with a polymer fiber length of 1.6 mm and the modes taking part in interference.

The light-guiding property of the POFI was experimentally investigated by a polarization controller (PC) connection. The input light from an ASE (Amplified Spontaneous Emission) light source (1250–1650 nm, from Fiber Lake Co., Ltd., Shenzhen, China) was send to the POFI through a PC, which is used to adjust the polarization component of input light. The output signal was detected by an optical spectrum analyzer (OSA, YOKOGAWA, AQ6370C). Figure 4a shows the transmission spectra of one POFI with a polymer fiber length of 0.8 mm and a section-size of 1.0 × 1.6 μm^2^. When the polarization of input light was adjusted to perpendicular to the major axis of the POF cross section, by rotating the half-wave plate of the PC, it shows a large insertion loss of ~−18 dB at 1360 nm, and no obvious interference pattern can be found as black line shown (low contrast). When the polarization of input light was adjusted to parallel to the major axis of the POF cross section, it exhibited a much lower insertion loss of ~−5 dB at 1360 nm, and an apparent interference spectrum can be obtained as red line shown (high contrast). It can be shown that the high-order mode with perpendicular polarization has large transmission loss and the coupling loss is mainly resulting from the mode field mismatch between TCF and POFI. To reduce the FSR, a longer sample (~1.6 mm) with the same fiber dimension has been fabricated and its transmission spectrum, shown in Figure 4b, where the FSR is decreased to be 45 nm. The modes which make interference are simulated by finite element method, and the simulated mode fields are shown in Figure 4b. The corresponding mode effective refractive index is 1.39 (@LP_01_) and 1.36 (@LP_11_). The simulated result is in good agreement with the measured FSR.

**Figure 5:**
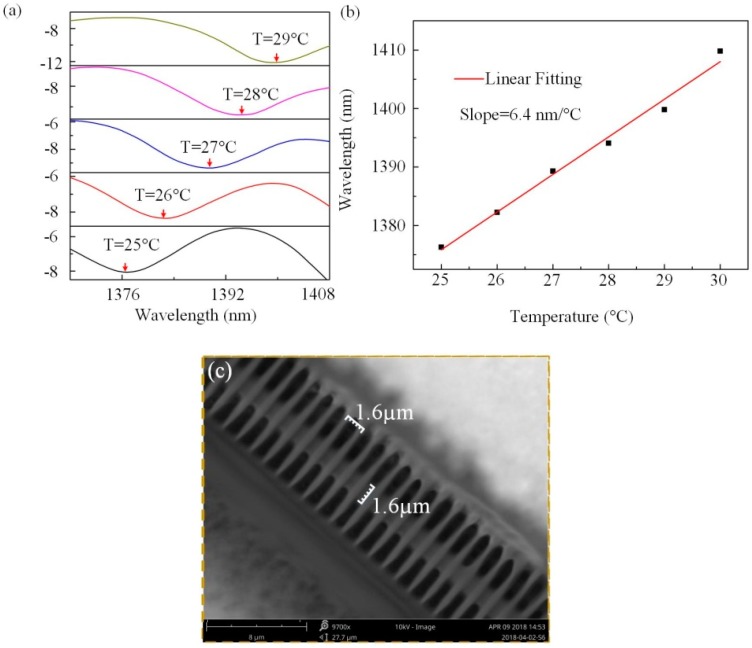
(**a**) Transmission spectral evolution of the POFI as the temperature is increased from 25 °C to 30 °C. (**b**) Dip wavelength versus temperature from 25 °C to 30 °C. (**c**) Scanning electron microscope (SEM) image of the POFI.

The temperature response of the POFI has been investigated using a controllable furnace. The supported grating (grating pitch = 1.6 μm; pitch number = ~1100) is microprinted perpendicular to the rectangular POF. An interference dip (@1376.3 nm at 25 °C) was used to monitor temperature changes in the experiment. The temperature was gradually increased from 25 °C to 30 °C with a step size of 1 °C, and maintained for 15 min at each step. Figure 5a demonstrates the spectral evolution of transmitted light as the ambient temperature was increased. The clearly red-shift in the interference dip marked by arrows is clearly evident in this process. Figure 5b shows the linear fitting relationship between the dip wavelength and ambient temperature. There is a small deviation between the measured data and the fitting line that may be caused by measurement error of the interference dip. It is noted that a high temperature sensitivity of ~6.4 nm/°C is achieved by the high thermal expansion of the polymer that induces extra OPD between high-order and fundamental modes. In the experiment, the temporal response was estimated as millisecond, which is similar to the results in Reference [24]. Figure 5c shows the scanning electron microscope (SEM) image for the sample with a polymer width of 1.6 μm and a gating pitch of 1.6 μm. The glass transition temperature (T_g_) of the used resin is ∼100 °C, which is demonstrated in the Sartomer product manual. Experimentally, the maximum operating temperature of this device is found to be 80 °C.

From Equation (2), the wavelength drift is proportional to the change of refractive index difference, and thermal-optical effect and thermal expansion effect both contribute to the material refractive index change when the ambient temperature is rising. In general, the thermal-optical coefficient of polymer is a negative value resulting in the spectral blue shift as temperature rises and the temperature response reported is ~−0.1 nm/°C [25,26]. Thus, we believe thermal expansion effect plays a main role for the wavelength drift in this experiment. The difference of the mode effective refractive index will increase because of the broadening of the POF dimension caused by thermal expansion. However, as the thermal expansion coefficient of polymer material is mostly on the order of 10^−4^/°C, the temperature sensitivity is mainly due to the optical path difference between fundamental mode and high-order mode. Thus, temperature sensitivity can be improved by increasing the length and cross-section ratio of the cured polymer waveguide.

## 4. Conclusions

Fs laser induced MPP has been successfully employed to fabricate an ultracompact POFI, embedded in a HCF for temperature measurement. Such a method offers broad tuning of the waveguide diameter and length. To realize the suspended POF configuration, the PR with a strong mechanical property has been proposed and the fabrication process has been introduced. The high-order modes mainly propagate in a certain direction due to the rectangular-like cross-sectional shape. Thus, its light-guiding properties were explored by means of a PC connection. Finally, a linear thermal response was achieved with a high sensitivity of 6.4 nm/°C within the temperature range from 25 °C to 30 °C, which is much higher than common fiber temperature sensors. In real application, to avoid the humidity influence, the POFI can be inserted into a section of glass tube having an interior diameter of 250 μm and the glue is applied to seal the device. Such a highly integrative POFI offers a new design route for fiber sensor systems, as well as optical fiber integrative micro-systems with multi-functions. In sum, the novel method has laid a foundation work for subsequence research on complex fiber-integrated polymer system—even in fiber chip integration technique.
